# Evaluating immune response *in vitro* in a relevant microenvironment: a high-throughput microfluidic model for clinical screening

**DOI:** 10.37349/etat.2022.00117

**Published:** 2022-12-29

**Authors:** Flora Doffe, Layla Fuoco, Judith Michels, Sandra Jernström, Raphael Tomasi, Pierre Savagner

**Affiliations:** 1 INSERM U1186, Integrative Tumor Immunology and Immunotherapy, Gustave Roussy, Faculty of Medicine, University Paris-Saclay, 94805 Villejuif, France; 2 Okomera, iPEPS, Pitié-Salpêtrière Medical center, 47 Hôpital Blvd, 75013 Paris, France; 3 Oncological Medecine Department, Gustave Roussy, 94805 Villejuif, France; University of Salford, UK

**Keywords:** Microfluidic model, personalized medicine, breast cancer, resistance to treatment, immune response

## Abstract

**Aim:**

Functional screening of new pharmaceutical compounds requires clinically relevant models to monitor essential cellular and immune responses during cancer progression, with or without treatment. Beyond survival, the emergence of resistant tumor cell clones should also be considered, including specific properties related to plasticity, such as invasiveness, stemness, escape from programmed cell death, and immune response. Numerous pathways are involved in these processes. Defining the relevant ones in the context of a specific tumor type will be key to designing an appropriate combination of inhibitors. However, the diversity and potential redundancy of these pathways remain a challenge for therapy.

**Methods:**

A new microfluidic device developed by Okomera was dedicated to the screening of drug treatment for breast cancer. This microchip includes 150 droplet-trapping microwells, offering multi-chip settings and multiple treatment choices.

**Results:**

After validating the system with established cell lines and a panel of drugs used clinically at Gustave Roussy, preclinical experiments were initiated including patient-derived xenograft (PDX) and primary tumor cells-derived tumoroids with the collaboration of Gustave Roussy clinicians. Tumor-isolated lymphocytes were also added to the tumoroids, using secondary droplets in proof-of-concept experiments.

**Conclusions:**

These results show the relevance of the methodology for screening large numbers of drugs, a wide range of doses, and multiple drug combinations. This methodology will be used for two purposes: 1) new drug screening from the compound library, using the high throughput potential of the chip; and 2) pre-clinical assay for a two-weeks response for personalized medicine, allowing evaluation of drug combinations to flag an optimized treatment with potential clinical application.

## Introduction

More than 50% of cancer patients show innate or acquired resistance to their first treatment over time [1]. Personalized cancer treatment aims to improve these response rates and focuses primarily on genomic approaches. However, the majority of the most effective treatments do not rely on exploiting tumor gene mutations [2]. In addition, new and more relevant study models are in great demand for the development of therapeutic molecules in oncology. Indeed, 80% of drugs validated by *in vitro* or mouse model studies fail before reaching clinical trials since these models do not recapitulate faithfully enough the tumor microenvironment structure and composition [3]. The two-dimensional (2D) *in vitro* models are by far the most widely used assays since they can be easily integrated into high-throughput screening processes of pharmaceutical compounds. Nevertheless, these models face serious limitations. In particular, they do not allow the reproduction of the three-dimensional (3D) microenvironment of a tumor, which is essential to control the effectiveness of treatment and potential interactions with immune cells [4].

The ideal model for high-throughput screening would be an *in vitro* model incorporating a relevant cellular and physical microenvironment, designed to mimic the tissue organization of a particular primary tumor at a specific stage of tumor progression. It should also be possible to recreate cell-cell and matrix-cell interactions. This model should also offer a large screening capacity to test the different signaling pathways, and the activity of therapeutic molecules as single agents or in combination to bring concrete results appropriate for clinical applications in a sufficiently short time. In addition, it should allow the sequential addition of drug treatments and immune cells [5] to allow the evaluation of the anti-tumor immune response and the response to immunotherapy. Finally, this model should allow the monitoring of essential cellular responses, including (but not limited to) cell death, proliferation, invasiveness, and immune response.

Recently, microfluidic approaches such as organ-on-chip or tumor-on-chip have emerged in numerous publications. They allow tumor cells to be injected directly into a miniaturized chip. These devices favor the development of complex structures from co-cultures of cells, all in a limited volume, a very promising approach in the field of precision medicine, where the size of the available biopsy or surgical sample is limited. Nevertheless, most commercial chips are not designed for arrays, only including a few tumoroids per chip, far from the conditions required to test a whole library of treatments [6]. The microfluidic tool developed by Okomera offers the ability to simultaneously monitor several hundred tumoroids developed from a single biopsy in real time, all on a single set of chips [7]. These chips are designed for multiplexing to test, for example, a library of treatments, thus offering the means to apply high-throughput screening. Moreover, it is possible to recreate a 3D tumor microenvironment, thus conferring the advantage to test a clinical sample within a stable and controlled environment for one week. Finally, this model does not require a large number of tumor cells, an essential feature considering the limited amount of biological material available during a biopsy.

## Materials and methods

### Chip design

The chip design is shown in the results section and described in Tomasi et al. [7]. In this case, there were 154 anchors disposed along a hexagonal pattern in the 2 cm^2^ trapping chamber. Anchors are 165 mm high.

### Experimental microfluidic protocol

The cell suspensions were loaded in glass (SGE, Trajan, Ringwood Australia) or plastic (Terumo, Tokyo Japan) syringes, that were actuated with programmable and computer-controlled syringe pumps (neMESYS, Cetoni, Korbusen, Germany). The syringes were directly connected to the polydimethylsiloxane chips with polytetrafluoroethylene tubing (Adtech, Gloucestershire England). For the merging of droplet pairs, the trapping chambers were perfused with a 20% (v/v) 1*H*,1*H*,2*H*,2*H* perfluoro-1-octanol (Sigma-Aldrich, Saint-Quentin-Fallavier, France) solution dissolved in Novec™-7500 engineered fluid (3M™, Cergy-Pontoise France, 3 mol/L).

### Spheroid formation on chip

The chips were first filled with a 3% (w/w) fluorosurfactant solution. All air bubbles were eliminated. MCF7 cells [American Type Culture Collection (ATCC), HTB-22] were detached from the culture flasks with a 5 min incubation in trypsin 0.5% ethylenediamine tetraacetic acid (Gibco, Asnières-sur-Seine, France, 25300), inactivated with complete medium. The cells were centrifuged at 1,200 rpm for 5 min and the cell concentration was determined using a hematocytometer (Neubauer hematocytometer). The cell pellet was resuspended (1 × 10^6^ cells/mL) for direct use. One glass syringe was loaded with this solution and droplets were produced according to the flow rates in the Dulbecco’s modified Eagle medium (DMEM, Gibco, 41965-039). The flow delivered the droplets to anchor wells where they quickly settled. After the loading, the chips were kept immersed in Milli-Q water in the CO2 incubator. Cells settled and regroup at the bottom of each droplet when the flowrates were stopped, organizing functional spheroids.

### Droplet library production with fluo barcodes

Droplet libraries are formed according to the protocol published by Tomasi et al. [7]. First, a segmented flow is produced inside a microtubing by alternatively aspirating a microliter volume of one of the different aqueous solutions to be tested and oil. This segmented flow is injected inside a microfluidic chip where a gradient of confinement transforms each aqueous segment into nanoliter droplets. These droplets are randomly mixed and injected into the main droplet array where they get trapped alongside the cell droplets. Two barcodes were designed. The first one was designed with 10 conditions using 3 fluorescent dyes at 4 concentrations, then mixed in a combinatorial manner to obtain 10 barcodes. CF™647 hydrazide and CF™488A hydrazide were used at 20.0 μmol/L, 2.5 μmol/L, 0.32 μmol/L, and 0.04 μmol/L. CF™350 hydrazide was used at 300 μmol/L and 50 μmol/L. The signals for these 3 dyes are respectively represented in the results section 1 in red, green, and blue. The second barcode used 5 conditions depicted in the results section.

All solutions were prepared with dimethyl sulfoxide [DMSO, 0.8% (v/v), D8418], propidium iodide (PI) 0.3 μmol/L (Sigma, Saint-Quentin-Fallavier France, P4864). Each barcode solution of 100 μL was aspirated sequentially, with 1 mL of fluorinated oil in between each plug. These fractions were injected in a “droplet-production” chip with specific geometrical parameters, an injector width and height respectively of 100 mm and 40 mm and a slope of 8%. The volume of the first (with the cells) and second (with the drug) droplets was respectively estimated to 50 nL and 14 nL, so the dilution factor from the library to the post-merging droplets was approximately 3.5.

### Image analysis

A custom “Matrix laboratory” code (R2016a, Mathworks) allowed us to detect each anchor and compute the red-green-blue values in the center of each droplet, before and after merging. For the droplet fluorescent barcode assignment and the toxicity experiments, single images of the anchors were acquired automatically with the motorized stage of the microscope. The analysis was conducted on an array of the detected anchors using a protocol previously described [8]. Briefly, cells were detected using bright fields and fluorescent intensities, and spheroids were selected based on morphological parameters. For each spheroid, the local background was used to determine a specific threshold for the fluorescent dead cells. The viability at the spheroid level was then defined being respectively the number of dead pixels and the area of the spheroid.

### Cell culture

MCF7 cells were grown in DMEM supplemented with 2 nmol/L beta-estradiol (Sigma, Saint-Quentin-Fallavier France, E-2758), 10% fetal bovine serum (Gibco, A3840401), and 1% penicilline/streptavidine (Gibco, 15140122). Cells were routinely subcultured at split ratios of 1:5. MCF-10A cells were also purchased from the ATCC (CRL-10317; Manassas, VA, USA) and grown in DMEM/Ham’s F12 (Gibco, 31331) containing 5% horse serum (Sigma, Saint-Quentin-Fallavier France, H1138), 20 ng/mL epidermal growth factor (Sigma, Saint-Quentin-Fallavier France, E4127), 0.5 μg/mL hydrocortisone (Sigma, Saint-Quentin-Fallavier France, H0888), 0.1 g/mL cholera toxin (Sigma, Saint-Quentin-Fallavier France, C8052), 10 μg/mL insulin (Sigma, Saint-Quentin-Fallavier, France, 19278) and 1% penicilline/streptavidine solution.

### Patient-derived xenograft processing

Patient-derived xenograft (PDX)_BRE-IGR-0134 primary cells originate from a docetaxel-treated breast luminal B (LumB) tumor grade 3, stage pT4, stage pN3, estrogen receptor and human epidermal growth factor receptor 2 negative. It was transplanted and left to grow until reaching 1,500 mm^3^ on 3 passages by the preclinical evaluation platform at Gustave Roussy. The tumor was collected and processed in our laboratory. Tumor dissociation was adapted from Corgnac et al. [9]. Upon arrival, the tumor tissues were weighed and washed with phosphate-buffered saline. The tumor was cut to isolate two random pieces of 1–3 mm^3^ that were fixed in formalin for histopathological analysis and immunohistochemistry, for 24 h at 4°C. The remaining tissues were minced in a Petri dish with a scalpel and directly digested in an enzyme mix (Tumor Dissociation Kit human, Miltenyi, Bergisch Gladbach, Germany, 130-095-929). The samples were incubated in a 37°C incubator under continuous rotation for 40 min. Evaluation of the viability was carried out with trypan blue. If the sample was necrotic, dead cells were removed by magnetic sorting (dead cell removal kit, Miltenyi, 130-090-101) following the manufacturer’s instructions. Live cells were kept in a supplemented medium (Supplementary materials), but experiments were performed in the absence of growth factors and inhibitors. The simplified medium is composed of DMEM, 10% fetal bovine serum, 1% penicilline/streptavidine, and 0.8 nmol/L beta-estradiol.

### Ovarian tumor preparation

Patients’ samples are collected within the established Ovbiomark biomarker trial (ID-RCB N: 2015-A01183-46). The protocol was submitted to the Ethic Committee/Institutional Review Board/People’s protection committee, which gave its approval on June 7th, 2016. The competent authority has approved the protocol on May 12th, 2016. The medium for the culture of ovarian primary cells with ovarian TumorMacs™ supplement (Miltenyi, 130-119-480) 1 mL in TexMACS™ medium (MACS media Miltenyi, 130-097-196). We sorted with magnetic beads tumor cells (Miltenyi, 130-108-339) and CD8+ lymphocytes (Miltenyi, 130-121-560) from the primary tumor. Then we loaded tumor cells at 2 × 10^6^ cells/mL in the chip, left them self-organize for 48 h, and then added a second droplet including the CD8+ population according to the protocol [9]. We cultured the CD8+ cells during that 48 h with the RPMI 1640 (Gibco, 61870-010) with 10% serum human group AB (Biotechnology Institute Jacques Boy, France, 201021334), 1% sodium pyruvate (Gibco, 11360-039), 0.2% penicilline/streptavidine filtered with Millipore 0.22 μm size (Sigma, Saint-Quentin-Fallavier France, S2GPT01RE) supplemented with 50 units of interleukin-2 (IL-2, Miltenyi Biotec, 130-097-744). After 48 h we added the third drop for treatments. We used a CF™647 hydrazide barcode at 2.0 μmol/L for the sample with CD8+ cells labeled with carboxyfluorescein diacetate succinimidyl ester (CFSE, Sigma, Saint-Quentin-Fallavier France, SCT110). The control sample without immune cells had no barcode. In all cases, we included PI at 0.3 μmol/L. Pictures were taken at 1 h, 24 h, and 48 h.

### Drug treatment

For MCF7 experiments, alpelisib (Medchem Express, HY-15244) was used at 1 μmol/L, 8 μmol/L, and 63 μmol/L; tamoxifen (Sigma, Saint-Quentin-Fallavier France, T5648) was used at 0.5 μmol/L, 4 μmol/L, 30 μmol/L, and 250 μmol/L. For PDX experiments, alpelisib was diluted at 0.1 μmol/L, 1 μmol/L, 8 μmol/L, and 63 μmol/L and 4-OH-tamoxifen (BioGems, 6800637) was used at 0.06 μmol/L, 0.5 μmol/L, 4 μmol/L, and 32 μmol/L.

For PDX experiments, fulvestrant (Sigma, Saint-Quentin-Fallavier, France, I4409) was used at 0.4 μmol/L in all the conditions, and alpelisib was added at 0.1 μmol/L, palbociclib (Sigma, Saint-Quentin-Fallavier France, PZ0383) at 1.6 μmol/L and everolimus (Sigma, Saint-Quentin-Fallavier France, SML2282) at 16 nmol/L was prepared in 0.08% DMSO. Doses were based on our prior 2D assays and Gustave Roussy clinicians’ expertise. In all conditions, 0.3 μmol/L of PI was added to follow the cytotoxicity.

### Microscopy

For imaging, pictures and movies, an IX83 microscope (Olympus, Tokyo, Japan) was used with proprietary analysis software (CellSense Dimension, Olympus).

## Results

### Designing a clinically relevant workflow using Okomera’s microfluidic technology

Okomera has designed a droplet microfluidic approach for rapid 3D spheroid formation in aqueous droplets. It integrates both the benefits of microfluidics (droplet encapsulation, miniaturization, high-throughput, perfusion) and microarrays (immobilization, easy imaging) in a single chip including 154 traps [7]. Several chips can be loaded simultaneously, displaying an even larger number of tumoroids. Miniaturization enables testing a small number of tumor cells (7,700 cells/chip) and potentially microbiopsy-derived samples, a breakthrough approach that would allow repeated measurements in the case of tumor progression (Sart et al. [8]).

**Figure 1.**
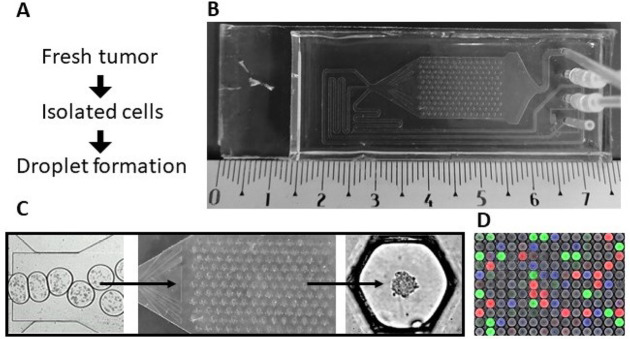
Microfluidic chip model (see details in Tomasi et al. [7]). A) Protocol from the tissues to the spheroids; B) Okomera chip; C) droplet formation in the hundreds of anchor traps, leading to spheroid formation; D) image analysis with a fluorescent barcode: blue (450 nm), green (488 nm) and red (555 nm)

**Figure 2.**
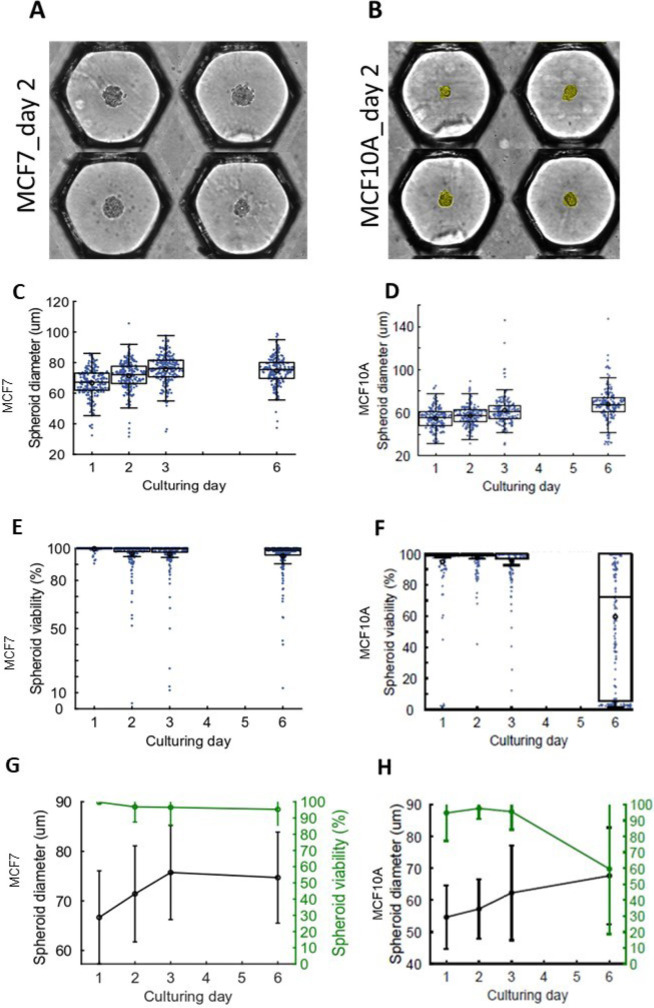
The 3D mammary breast cell lines LumB tumor MCF7 and epithelial MCF10A cells. (A) MCF7; and (B) MCF10A cells sediment into the anchoring traps in medium droplets and aggregate into a spheroid within 48 h. The yellow coloring shows the segmentation occurring during the analysis of the spheroid. Anchoring traps are shown side-to-side after image reconstruction for image analysis. Box dot plot showing diameter of (C) MCF7 and (D) MCF10A spheroids over time. Box dot plot of the viability of (E) MCF7 and (F) MCF10A over time. Comparison of the diameter *versus* viability changes over time for (G) MCF7 and (H) MCF10A

Tumor cells are injected as a single-cell suspension and are encapsulated in nanoliter aqueous droplets on the chip. The droplets flow through the distribution channel to the main compartment of the chip, where they are immobilized on an array of traps (Figure 1) [7]. The cells sediment at the bottom of the droplets and aggregate, forming a single spheroid per droplet in 24 h (Figure 1; Figure 2A, 2B).

We first used two human epithelial mammary cell lines to set the conditions: the mammary tumor cell line MCF7 (Figure 2A), expressing a tight luminal epithelial phenotype and the mammary immortalized cell line MCF10A (Figure 2B), expressing a loose basal phenotype, more appropriate for morphogenic studies. Both cell lines formed spheroids. They were resuspended to obtain 50 cells in 50 nL, the average volume of the drop in the microchip. We cultured the MCF7 cells for 6 days and observed a significant spheroid growth, estimated by the spheroid diameter (Figure 2C). We also cultured MCF10A cells and could monitor slower growth (Figure 2D). Noticeably, there was no increase in mortality for MCF7 cells during this period (Figure 2E). By contrast, MCF10A spheroids started to decay after 6 days (Figure 2F). In conclusion, MCF7 cells grew and survived better in the microchip environment than MCF10A cells (Figure 2G, 2H).

We then used the double anchor design [7] to initiate the sequential addition of secondary droplets, followed by droplet pair merging. By implementing a triangle on the anchor shape, two distinct areas of different trapping forces are created on a single anchor: one strong trapping area capable of immobilizing large droplets (the circular part), and one weaker trapping area only capable of immobilizing small droplets (the triangular part). Therefore, reproducible droplet pairs can be immobilized in the microfluidic chamber. Droplet pairs can be easily merged by perfusing a destabilizing reagent in the external oil. This droplet pairing technology enables the introduction of new components such as drugs, growth media, staining, etc., to the original spheroids.

**Figure 3.**
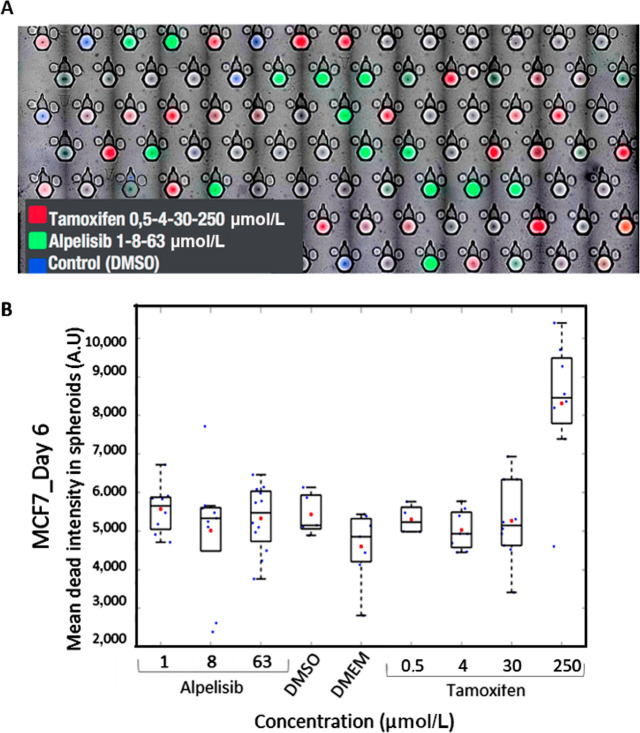
Treatment of MCF7 tumor cells in the microchip. A) Microscopy image of 5 merged channels: brightfield (BF, detect the trap and the spheroids), 450 nm (blue) DMSO control, 488 nm (green) alpelisib, 647 nm (red) tamoxifen, cytotoxic dye PI at 555 nm; B) cytotoxic (PI) mean dead intensity resulting from different dilution of alpelisib and tamoxifen at day 6

**Figure 4.**
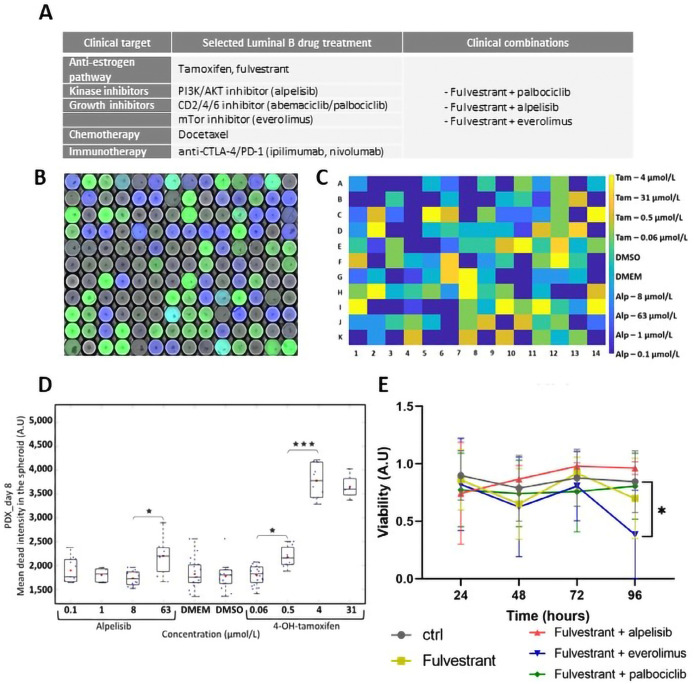
Treatment combination on LumB PDX. A) List of selected clinical treatments, including combinations used clinically; B) raw composite image combining 450 nm, 488 nm, and 647 nm analysis provides the key to identifying treatment-associated fluorescent labeling; C) in another experiment, barcode processing and image analysis provide the location for individual treatment for each well; D) cytotoxicity evaluated in PDX at day 8 for alpelisib and 4-OH-tamoxifen; E) viability over time after fulvestrant and combined treatments. Blue: control DMSO, orange: fulvestrant, yellow: fulvestrant + alpelisib, violet: fulvestrant + everolimus, green: fulvestrant + palbociclib. Statistical analysis: two-way ANOVA. * *P* < 0.01. CTLA-4: cytotoxic T-lymphocyte-associated antigen 4; PD-1: programmed cell death protein 1

The sequential addition of droplets with different contents, treating each spheroid individually, enables multiple testing of the spheroids. The sequential drops are barcoded with fluorophores and can be tracked by imaging. This technology enables the cultivation of tumor cells, to test their sensitivity to chemo/immunotherapies by adding sequential droplets to the trapped droplets (Figures 3 and 4).

### Testing tumor cell lines: proof of concept experiments

We tested several drugs targeting breast cancer on the tumor cell line MCF7. We selected tamoxifen, an anti-estrogen drug used frequently for luminal breast tumors, known to express estrogen receptors and alpelisib an anti-kinase inhibitor used in personalized therapy (Figure 3). The cells were loaded in the microchip for 24 h. Then the drug library including the fluorescent bar coding was added in a second drop. The bar coding allowed identification treatment for each trap by image analysis (Figure 3A).

Observation at different wavelengths provided the keys to the bar code and the cytotoxicity extent, using 4 channels and BF imaging to locate spheroids: 450 nm (blue) DMSO control, 488 nm (green) alpelisib, 647 nm (red) tamoxifen, cytotoxic dye PI at 555 nm. As expected, tamoxifen did not induce cell death in tumor cells other than the highest dose tested (250 μmol/L). Alpelisib is a growth inhibitor and did not induce cell death (Figure 3B). The dose range for tamoxifen went from 1× to 8×. This proof-of-concept experiment demonstrated the potential of the system for dose-response experiments.

### Testing primary tumor cells derived from PDX

Once loading and treatment conditions had been standardized, we introduced patient-derived cells, isolated from a LumB tumor PDX. Tumors were dissociated to obtain a suspension of 50 tumor cells per 50 nL. We still focused on LumB breast cancer and chose several drugs used clinically, alone or combined for LumB breast cancer patients (Figure 4A).

We designed a library (Figure 4A) based on 4-OH-tamoxifen a metabolite that shows better affinity to the receptor estrogen in cell culture. We used a lower concentration, between 0.06 μmol/L and 32 μmol/L. After barcode processing and image analysis (Figure 4B, 4C), we could detect a cytotoxic impact on the PDX’s at 4 μmol/L with 4-OH-tamoxifen, but no cytotoxic effect with alpelisib (Figure 4D). We then tested another library combining other drug treatments with 4-OH-tamoxifen (data not shown) and including fulvestrant, another anti-estrogen used clinically, more specific than the tamoxifen. After image processing, we determined that the combination fulvestrant and everolimus were the most efficient to induce cell death at 96 h (Figure 4E).

### Primary tumor cells from biopsy and immune cells introduction: cytotoxicity testing

**Figure 5.**
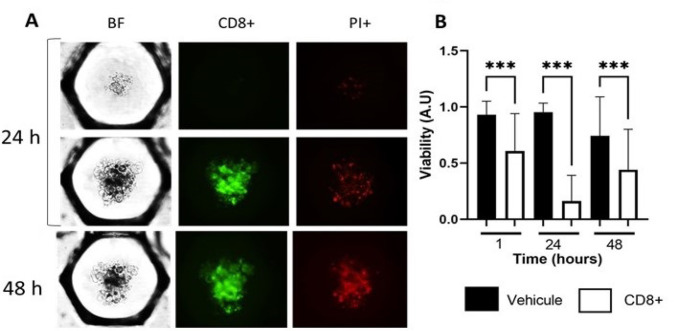
Quantifying CD8+ lymphocyte-induced cytotoxicity in primary ovarian cancer. A) Ovarian primary tumor cells with and without CD8+ at 24 h and 48 h, BF for the detection of the spheroids, CFSE (green) CD8+, 555 nm PI dead cells; B) boxplot of the viability with and without CD8+ at 1 h, 24 h and 48 h. Statistical analysis: two-way ANOVA. *** *P* < 0.001

A major hurdle for immune response evaluation in a tumor is to quantify cell-mediated cytotoxicity in a relevant 3D *in vitro* model. Cytotoxic CD8+ and natural killer (NK) lymphocytes are key cell populations of the immune system that can locate and efficiently eradicate tumor cells. They are essential for immune response in LumB. For the proof of concept, we have used a primary ovarian tumor after the dissociation of the tissue and retrieval of lymphocyte T CD8+ population infiltrated within the tumor. After 24 h spheroid assembling, CFSE-labeled primary lymphocytes were added in a second drop with a ratio of ten CD8+ T cells for one tumor cell (Figure 5A). Cytotoxicity was monitored after 24 h and 48 h. Lymphocyte-induced cell death mostly occurred during the first 24 h. However, they were still able to induce significant tumor cell death at 48 h (Figure 5B). We verified by microscopy that lymphocytes (CFSE+) were not accounting for the PI+ dead cells, confirming that the quantification of the PI signal reflected the tumor cell death in the absence of fluorescent cross talk.

## Discussion

Reflecting the poor clinical relevance of classic 2D culture drug screening strategies, the use of 3D methodology and organoids for pre-clinical applications has become a leading trend. Microphysiological systems become a key component in basic research and pharmacological projects [4–6]. They have been improving significantly, decreasing the number of animal models used for the first stage of preclinical trials. Various systems are competing: most recent strategies focus on multi-organs culture and interactions using connected chips and creating a near-physiological platform [10, 11].

Accordingly, more than 150 ongoing clinical trials based on spheroids or organoids are now cited in the reference site ClinicalTrials.gov (August 2022). Unfortunately, most clinically relevant chips and designs appear poorly adapted for large throughput screening [12]. Assays designed for large throughput screenings mostly rely on 3D spheroids, providing relatively fast survival data [13–15], using drug doses based on blood titers determined in treated patients. Clearly, these models provide faster results than *in vivo* models. However, they do not involve drug metabolization in most cases, promoting the use of hydroxy-tamoxifen in our 3D models for example, as the active metabolite for tamoxifen. Also, the question of a relevant culture medium remains open, as to how to avoid animal-derived components in culture media and matrix when organoids are used in 3D models.

Considering the ground-breaking progress linked to cancer immunotherapy, designing co-cultures introducing and testing immune cells with tumoroids becomes a requisite. Several recent publications show that introducing immune cells like NKs can be efficient in targeting breast cancer tumoroids [16], or in pediatric malignancies when used in combination with treatments [17]. In non-tumoral models, immune cells have already been co-cultured with gut and liver cells separately using transwell devices [18], providing detailed information on cell characterization, and gene and cytokine expression levels. However, none of these reports used a high throughput method. Others have been using similar chip designs to study immune cells on commercial tumor cell lines and cardiac spheroids [19]. However, to the best of our knowledge, none of these publications involved autologous tumors and immune cells. By today’s standards, it appears as a necessity to introduce autologous immune cells to gain clinical relevance. Lymphocytes or NKs that infiltrate the tumors can be directly combined with drug treatment to monitor immune response.

Another potentially important component is stroma cells such as fibroblasts. It is known that cancer-associated fibroblasts have an impact on tumor progression, for example in ovarian cancer [20] and that the culture and co-cultures in 2D and 3D show at a genomic and proteomic level significant differences in liver cancer models [21]. Introducing stroma cells brings a new level of complexity, but will most likely have to be integrated for clinical relevance.

One direct goal for 3D models is to become a routine tool for personalized medicine. Predicting the response to treatment before prescribing it to patients is the goal today. In terms of timing, using a biopsy to generate a resistance chart in a week would directly support the therapy decision process. The relevance of the method is now supported by PDX experiments. Chakrabarty et al. [22] have shown that tumor-derived PDX provided a reliable predictive tool to anticipate the effect of treatment on tumors, in that case, to assess resistance to cisplatin in breast tumors. The limit for this approach is the amount of time necessary to grow the PDX, amounting to several weeks in mice, to be added to the time necessary for drug screening. Unfortunately, this timing is not compatible with the process of clinical decision-making.

In this manuscript, we propose a new, fast and high-throughput method to test various treatments and combinations for personalized medicine assays. Okomera’s microfluidic chip miniaturizes and automatizes current manual and cumbersome workflows used for spheroid research. Indeed, pipetting robots do not adapt easily to 3D cell culture, where the 3D tissues are in suspension in non-adherent wells. Since it operates with nanoliter volumes, compared to the regular micro/milliliter scale for multiwell plates, the microfluidic chip enables a 1,000-fold volume reduction, providing high throughput (150 traps/chip, 8 chips per experiment) and multiplexed testing even when there is only a small amount of material available, the most common case in cancer biopsies. The miniaturization also leads to shortened experimental times as the cells, in much closer proximity in a nanoliter droplet than in a well of a plate, aggregate faster (within 24 h) to form 3D spheroids that can evolve in organoids when matrix elements are introduced. Unlike commercially available organs-on-chips systems that only enable testing a single condition per chip, Okomera’s droplet encapsulation enables testing multiple conditions on one single chip. This chip format, in the range of a microscope slide, is compatible with most microscopy systems. It uniquely integrates the benefits of droplet encapsulation (high throughput, homogeneous parallelization, and multiplexing), microarrays (simple high throughput imaging of immobilized droplets), and microfluidic chambers (perfusion, immune-staining protocols on chip) [8]. The droplet pair merging enables biological applications such as 3D co-cultures, the addition of matrix elements to organize a hydrogel, and drug testing (Tomasi et al. [7]). The addition of a 3D matrix and mesenchymal cells will be a requirement to produce relevant tumoroids mimicking the original LumB organization. For LumB, hyaluronans/collagen appears as the most relevant practical choice [16].

In this report, we present the analysis of immune and treatment response in a new resistance model using cell lines, PDX cells, and primary ovarian tumor cells. We established a list of drug treatments routinely used for breast cancer patients at Gustave Roussy hospital. We first analyzed the growth of the tumor and epithelial cell lines in our microfluidic microchip. We could assess that the MCF7 tumor cell line grew faster than the MCF10A cells. They reached their maximal size in 3 days, maintaining good viability. Conversely, it took an average of 6 days for MCF10A cells to reach the same size. At this stage, cell viability was significantly altered, supporting the stronger resilience of tumor cell MCF7.

Because chemo and immunotherapy are not recommended in the first line for the treatment of LumB breast cancer, known to be estrogen-dependent, we used tamoxifen, an estrogen receptor inhibitor commonly used clinically and anti-kinase inhibitors, increasingly used in personalized medicine like alpelisib [23]. We found after one week of treatment that tamoxifen [24] was effective, but not alpelisib to induce cell death in the tumoroids in the microchip. This demonstrates the potential of these microchips to monitor the resistance of tumor cells to various drugs. We found out that a low dose of fulvestrant alone does not induce cell death in our PDX, nor when combined with palbociclib [25] or alpelisib. In this case, we found that fulvestrant + everolimus [26] was the most potent treatment for PDX-derived breast cancer cells after 4 days of treatment. Our large throughput approach opens the way to multiple treatments, including drug combinations, found to be potentially much more efficient for therapy. The monitoring of more specific cellular responses such as apoptotic pathways, necrosis, etc., will bring valuable information on the drug’s functional impact at the cellular level.

Finally, our model is particularly appropriate to introduce immune cells in direct contact with tumor cells and monitor immunotoxicity in real-time for 48 h. Over this period, CD8+ cells were mostly active in killing tumor cells during the first 24 h. However, they maintained cytotoxicity for 48 h, with a lowered impact. The present study focused on autologous CD8+, an appropriate choice for ovary cancer cells. Following these proofs-of-concept experiments, we will now look for the effect of an immunotherapy anti-PD-1 or anti-CTLA-4 [27] to monitor the impact on tumor cell immunotoxicity after adding autologous lymphocytes. This will be very helpful to determine which patient could respond or not to immunotherapies, an essential question for clinicians.

Taken together, our work supports a new screening method to be used for early resistance screening, in breast cancer. It is intended to be used after biopsy or surgery when the knowledge of the potential resistance to drugs and the immune response is particularly important for therapy decisions. Further work including a predictive clinical trial will be essential to validate the method.

## Abbreviations

2D: two-dimensional
3D: three-dimensional
BF: brightfield
CFSE: carboxyfluorescein diacetate succinimidyl ester
DMEM: Dulbecco’s modified Eagle medium
LumB: luminal B
NK: natural killer
PDX: patient-derived xenograft
PI: propidium iodide

## Supplementary materials

The supplementary material for this article is available at: https://www.explorationpub.com/uploads/Article/file/1002117_sup_1.pdf.

## Declarations

### Copyright

© The Author(s) 2022.
