# Supplementary material for: Evaluating immune response in vitro in a relevant microenvironment: a high-throughput microfluidic model for clinical screening
Source: Explor Target Antitumor Ther. 2022 Dec 29;3(6):853–65. doi: 10.37349/etat.2022.00117 (PMC9834268; doi:10.37349/etat.2022.00117)
Supplement: Supplementary file 1 [file etat-03-1002117-s001.pdf]

Table S1 Breast cancer organoid culture medium

**Components**	**Supplier**	**References**	**Final concentration**
DMEM/F12 + GlutaMax 1X	ThermoFisher	31331-028	1X
β-Estradiol	Sigma	E-2758	0,8µM
Hepes	Sigma	H3375	10 mM
Pen/Strep	ThermoFisher	15140-122	100 U/ml; 100 µg/ml
Primocin	InvivoGen	ant-pm-05	50 µg/ml
R-Spondin-1	Peprotech	120-38	5 ng/ml
Noggin	Peprotech	120-10C	100 ng/ml
FGF-7	Peprotech	100-19	5 ng/ml
FGF-10	Peprotech	100-26	20 ng/ml
EGF	Sigma	E4127	5 ng/ml
A83-01	Sigma	SML0788	500 nM
Y-27632 (ROCK inhibitor)	StemCell Technologies	72304	5 µM
SB202190	Sigma	S7067	500 nM
B27 supplement	ThermoFisher	17504044	1X
N-acetylcysteine	Sigma	A7250	1,25 mM
Nicotinamide	Sigma	72340	5 mM
Neuregulin 1 (NRG1-ß1)	Peprotech	100-03	5 nM
